# Molecularly guided treatment of metastatic parotid gland carcinoma in adults

**DOI:** 10.1007/s00508-020-01778-8

**Published:** 2020-12-09

**Authors:** Hossein Taghizadeh, Leonhard Müllauer, Robert M. Mader, Thorsten Füreder, Gerald W. Prager

**Affiliations:** 1 Department of Medicine I, Clinical Division of Oncology, Medical University of Vienna, Spitalgasse 23, 1090 Vienna, Austria; 2 Comprehensive Cancer Center Vienna, Vienna, Austria; 3 Clinical Institute of Pathology, Medical University Vienna, Vienna, Austria

**Keywords:** Molecular profiling, Molecular guided treatment, Precision cancer medicine, Head and neck cancer, Molecular oncology

## Abstract

**Background:**

Advanced therapy-refractory parotid gland carcinomas have a poor prognosis with limited therapy options. We used molecular profiling to offer molecular guided therapies to patients with advanced metastatic parotid gland malignancies.

**Methods:**

In this retrospective analysis we describe the molecular profiling of ten patients diagnosed with therapy-refractory metastatic parotid gland malignancies.

**Results:**

We identified seven genetic aberrations in five patients: two mutations in CDKN2A and one mutation in APC, ATM, TP53, SMARCB1 and FGFR1, respectively. No mutations were detected in five patients. The IHC demonstrated frequent expressions of EGFR and p‑mTOR, as well as PTEN in eight patients. For four fifths (*n* = 8) of the patients, a targeted therapy was suggested. Eventually, three patients received the targeted therapy recommendation and one patient achieved stable disease for 14 months.

**Conclusion:**

A total of eight therapy recommendations were provided. Based on our observations, molecular-guided therapies may be a feasible treatment approach for this rare disease entity.

## Introduction

Salivary gland carcinomas (SGC) comprise rare heterogeneous malignancies that account for only 5% of all head and neck cancers. The SGCs are classified into 24 subtypes according to the World Health Organization (WHO) definition. Likewise, the tumor biology and prognosis of SGCs markedly differ between histological types [1–4]. Among these glands, most malignancies occur in the parotid gland. The parotid gland carcinoma (PGC) is a relatively rare cancer, making up only 0.3% of all cancers combined [5]. The PGC with distant metastases, mainly in the lungs and bones, has a dismal median survival prognosis of 7.3 months despite therapeutic efforts [6].

The mainstay of treatment is complete surgical resection followed by postoperative radiotherapy (depending on the subtype and risk features). In surgical interventions, complete excision of the PGC is carried out with preservation of the functioning facial nerve, provided there is no tumor invasion. Systemic chemotherapy is generally indicated for patients with recurrent and/or metastatic PGC [5, 7–9].

The most common histological subtype in primary PGC is mucoepidermoid carcinoma (MEC) [10, 11]. Given the rarity of this disease, there are, apart from parotidectomy and radiotherapy, few well-established therapy standards for how to treat patients with progressive stage IV PGC [7].

There has recently been an effort to individualize therapy options in cancer diseases. In some instances, tailored therapy attempts with immunotherapeutics or tyrosine kinase inhibitors are used, e.g., trastuzumab in HER2-positive breast cancer or gastric cancer, imatinib in Philadelphia chromosome-positive chronic myeloid leukemia (Ph + CML), BRAF-directed therapy with vemurafenib or dabrafenib/trametinib in melanoma [12–14].

Emerging novel agents, such as the profiling of tumor molecular alterations and mutations as well as the identification of druggable targets and the ground-breaking pilot trial by von Hoff et al. have ushered in a new era of medicine; this approach has received many titles, such as individualized, stratified, tailored, or precision cancer medicine [15]. The main rationale of PCM is to match a therapeutic agent to its corresponding target for precise tailored therapy fitting a specific patient, aiming to achieve a deep durable and sustainable response without damaging healthy cells and tissues. This matches the tailored “therapeutic dress” to the patient [16].

We conducted a retrospective subgroup analysis of our precision molecular register, exclusively focusing on patients with progressive PGC with no available standard treatment options. These patients had been enrolled and whose tumors had been profiled in our special PCM platform. We sought to map the molecular profiles of advanced, relapsed and therapy-refractory PGC to evaluate whether there are any aberrations that can be targeted by a tailored therapy.

## Material and methods

### Ethics, consent and permission

The study was conducted in accordance with the International Conference on Harmonization E6 requirements for good clinical practice and with the ethical principles outlined in the Declaration of Helsinki. All patients had to provide written informed consent before inclusion in our PCM platform. Furthermore, the institutional ethics committee has also approved this subanalysis (Nr. 1039/2017).

### Patients and design of the precision medicine platform

Patients with PGC who had progressed through all standard treatment options were eligible for inclusion in our platform for precision medicine, provided archival tissue samples were available. Patients had to have an Eastern Cooperative Oncology Group (ECOG) performance status of 0 or 1. Our platform for precision medicine is not a clinical trial, but intends to provide the possibility of a targeted therapy to patients where no active anti-tumor treatment is available.

### Tissue samples

Formalin-fixed, paraffin-embedded tissue from patients with advanced PGC that were refractory to all available standard treatment lines were sent to or retrieved from the archive of the Department of Pathology.

### Cancer gene panel sequencing

DNA was extracted from paraffin-embedded tissue blocks with a QIAamp Tissue KitTM (Qiagen, Hilden, Germany) and 10 ng DNA per tissue sample was provided for sequencing. The DNA library was created by multiplex polymerase chain reaction with the 161-gene next-generation sequencing panel of Oncomine Comprehensive Assay v3 (Thermo Fisher Scientific, Waltham, MA, USA). The panel includes driver mutations, oncogenes, tumor suppressor genes, and gene fusions. See supplementary information for complete list of the gene panel. The Oncomine Comprehensive Assay v3 was optimized for sequencing on an Ion Personal Genome Machine System (Thermo Fisher Scientific). The generated sequencing data were afterwards analyzed with the help of the Ion Reporter Software (Thermo Scientific Fisher). We referred to BRCA Exchange, ClinVar, COSMIC, dbSNP, OMIM and 1000 genomes for variant calling and classification. The variants were classified according to a five-tier system comprised of the modifiers pathogenic, likely pathogenic, uncertain significance, likely benign, or benign. This classification was based on the standards and guidelines for the interpretation of sequence variants of the American College of Medical Genetics and Genomics. The variants pathogenic and likely pathogenic were taken into consideration for the recommendation of targeted therapy.

### Immunohistochemistry

The IHC was performed using 2‑μm-thin tissue sections read by a Ventana Benchmark Ultra stainer (Ventana, Tucson, AZ, USA). The following antibodies were applied: anaplastic lymphoma kinase (ALK, clone 1A4; Zytomed, Berlin, Germany), CD20 (clone L26; Dako Omnis from Agilent Technologies, Santa Clara, CA, USA), CD30 (clone BerH2; Agilent Technologies, Vienna, Austria), epidermal growth factor receptor (EGFR, clone 3C6; Ventana), estrogen receptor (clone SP1; Ventana), human epidermal growth factor receptor 2 (HER2, clone 4B5; Ventana), HER3 (clone SP71; Abcam, Cambridge, UK), C‑kit receptor (KIT, clone 9.7; Ventana), MET (clone SP44; Ventana), NTRK (clone EPR17341, Abcam), phosphorylated mammalian target of rapamycin (p-mTOR, clone 49F9; Cell Signaling Technology, Danvers, MA, USA), platelet-derived growth factor alpha (PDGFRA, rabbit polyclonal; Thermo Fisher Scientific), PDGFRB (clone 28E1, Cell Signaling Technology), programmed death-ligand 1 (PD-L1, clone E1L3N; Cell Signaling Technology), progesterone receptor (clone 1E2; Ventana), phosphatase and tensin homolog (PTEN, clone Y184; Abcam) and ROS1 (clone D4D6; Cell Signaling Technology).

To assess the immunostaining intensity for the antigens EGFR, *p*-mTOR, PDGFRA, PDGFRB and PTEN, a combinative semiquantitative score for immunohistochemistry was used. The immunostaining intensity was graded from 0 to 3 (0 = negative, 1 = weak, 2 = moderate, 3 = strong). To calculate the score, the intensity grade was multiplied by the percentage of corresponding positive cells: (maximum 300) = (% negative × 0) + (% weak × 1) + (% moderate × 2) + (% strong × 3).

The immunohistochemical staining intensity for HER2 was scored from 0 to 3+ (0 = negative, 1+ = negative, 2+ = positive, 3+ = positive) pursuant to the scoring guidelines of the Dako HercepTestR from the company Agilent Technologies (Agilent Technologies, Santa Clara, CA, USA). In the case of HER2 2+, a further test with HER2 in situ hybridization was performed to verify amplification of the HER2 gene.

Estrogen receptor and progesterone receptor staining were graded according to the Allred scoring system [17] from 0 to 8 and MET staining was scored from 0 to 3 (0 = negative, 1 = weak, 2 = moderate, 3 = strong).

For PD-L1, the tumor proportion score was calculated, which is the percentage of viable malignant cells showing membrane staining.

Staining for ALK, CD30, CD20 and ROS1 was classified as positive or negative based on the percentage of reactive tumor cells but without graduation of the staining intensity. In ALK or ROS1 positive cases, the presence of a possible gene translocation was evaluated by fluorescence in situ hybridization (FISH).

All antibodies used in this study were validated and approved at the clinical institute of pathology and are used in routine IHC staining for clinical purposes. The antibodies have been validated, by proper positive and negative tissue controls and by non-IHC methods, such as immunoblotting and flow cytometry, to detect the respective epitope of the antigens. For the control, the use of the antibodies was optimized in terms of intensity, concentration, signal/noise ratio, incubation times and blocking. The negative control was conducted by omitting the primary antibody and by substitution of isotype-specific antibody and serum at the exact same dilution and laboratory conditions as the primary antibody to preclude unspecific binding.

For the positive control, the antibodies were shown not to cross-react with closely related molecules of the target epitope.

### Fluorescence in situ hybridization

The FISH was performed with 4‑μm-thick formalin-fixed, paraffin-embedded tissue sections. The following FISH probes were employed: ALK (2p23.1; Abbott, Abbott Park, IL, USA), RET (10q11; Kreatech, Berlin, Germany), PTEN (10q23.31)/centromere 10, and ROS1 (ZytoVision, Bremerhaven, Germany), 200 cell nuclei per tumor were evaluated. The cut-off level for an aberrant ALK, RET, and ROS1 FISH was ≥15% of cells with a split-apart signal. The PTEN FISH was considered positive for PTEN gene loss with ≥30% of cells with only one or no PTEN signals. A chromosome 10 centromere FISH probe served as a control for ploidy of chromosome 10.

### Multidisciplinary boards (molecular tumor boards for PCM)

After thorough examination of the molecular profile of each tumor sample by a qualified and competent molecular pathologist, the results and findings were reviewed in multidisciplinary tumor boards (MTB) that were held every other week. Members of the board included molecular pathologists, radiologists, clinical oncologists, biostatisticians, and basic scientists. The MTB recommended the targeted therapy based on the specific molecular profile of each patient. The targeted therapies included tyrosine kinase inhibitors, checkpoint inhibitors (e.g. anti-PD-L1 monoclonal antibodies), and growth factor receptor antibodies with or without endocrine therapy. The treatment recommendations by the MTB were prioritized depending on the level of evidence from high to low according to phase III to phase I trials.

If more than one druggable molecular aberration was identified, the MTB recommended a therapy regimen to target as many molecular aberrations as possible, with special consideration given to the toxicity profile of each antitumor agent and their potential interactions. Since all patients were given all available standard treatment options for their cancer disease prior to their inclusion in our PCM platform, nearly all targeted agents were suggested as off-label use. If the tumor profile and the clinical characteristics of a patient met the requirements of a clinical trial for targeted therapies that was conducted in our cancer center, patients were preferentially asked if they wanted to participate in this trial.

### Descriptive statistics

For data description, we used measures of central tendency including the mean and median. We also used the method of frequency distribution to delineate the characteristics of the PGC patients.

## Results

All ten patients diagnosed with progressive primary PGC were included in this analysis from our platform for precision medicine that has so far profiled over 600 patients with various advanced solid tumors. All PGC patients were Europeans. Five men and five women were diagnosed with five different histological subtypes of primary PGC. The subtypes were acinic cell carcinoma (*n* = 1), adenocarcinoma NOS (*n* = 3), adenoid cystic carcinoma (*n* = 3), carcinoma ex pleomorphic adenoma (*n* = 1), and primary squamous cell carcinoma (*n* = 2). The primary tumor location was the right side in six patients (60%) and the left side in four patients (40%).

At the time of molecular profiling, all patients had an advanced, therapy-refractory and relapsed PGC in stage IV with distant metastases, mainly in the bones and lungs. The whole cohort had undergone parotidectomy and radiation therapy. Four patients had also received prior chemotherapy: two patients were treated with carboplatin and paclitaxel, one patient received cisplatin and cetuximab, and another patient was given a CAP regimen consisting of cyclophosphamid, doxorubicin (trade name Adriamycin) and a platinum-based agent (usually cisplatin).

The median age at the time of initial diagnosis was 59.5 years, ranging from 27 to 82 years, and the median age at the time of molecular profiling was 63 years, ranging from 37 to 83 years (Table 1).

**Table 1:** Patient characteristics (*N* = 10)

Patient characteristics	Number
Median age at first diagnosis (years)	59.5
Median age at molecular profiling (years)	63
Men	5
Women	5
Histological subtypes of parotid gland carcinoma	5
Caucasian	10
Relapsed disease	10
Stage IV	10
Parotid gland carcinoma on the right side	6
Parotid gland carcinoma on the left side	4
Therapy recommendations	8

Of the ten tissue samples, five were from metastatic sites and five from the primary site.

In total, we identified seven molecular aberrations in five patients: two mutations in CDKN2A and one mutation in each of APC, ATM, TP53, SMARCB1, and FGFR1. No mutations were detected in five patients.

Expression of EGFR, *p*-mTOR and PTEN was detected by IHC in eight patients. The EGFR median score was 120, and 3 patients had a high EGFR score of between 200 and 300. The expression of *p*-mTOR was lower with a median score of 70, and 2 patients had a high *p*-mTOR score of between 200 and 300. Expression of MET and PDGFRA was detectable in six and five samples, respectively. MET expression was weak in four patients and moderate in one patient. One sample exhibited a strong MET expression. Less common expressions were observed for KIT and AR which were observed in three and two patients, respectively. The KIT expression was found to be weak in two samples and moderate in one sample, AR was moderately expressed in both patients with adenocarcinoma.

IHC and FISH were not performed in one patient due to insufficient tumor material.

For eight of the ten patients, a targeted therapy was suggested based on their individual molecular profile (Table 1). Androgen deprivation therapy (ADT), crizotinib, and cetuximab each were offered in two cases and imatinib and sunitinib were proposed in one case. We refer here to Tables 2 and 3 for the rationale of the therapy suggestions.

**Table 2:** Rational for therapy recommendations

Therapeutic agent (trade name)	Targets	Overview of current FDA approval in different entities	Overview of current EMA approval in different entities
Cetuximab (Erbitux) (*n* = 2)	EGFR	CRC, HNSCC	CRC, HNSCC
Crizotinib (Xalkori) (*n* = 2)	ALK, ROS1 MET overexpression	ALK or ROS1 positive NSCLC	ALK or ROS1 positive NSCLC
Imatinib (Gleevec) (*n* = 1)	PDGFR, KIT, Bcr/Abl	Ph + CML, KIT + GIST, MDS/MPD associated with PDGFR, Ph + ALL	Ph + CML, KIT+ GIST, MDS/MPD associated with PDGFR, Ph + ALL
Sunitinib (Sutent) (*n* = 1)	PDGFR, KIT, VEGFR, RET, FLT3	RCC, PDAC, GIST	RCC, PDAC, GIST

*ABL* Abelson murine leukemia viral oncogene homolog 1, *ALK* Anaplastic lymphoma kinase, *ALL* acute lymphatic leukemia, *BCR* breakpoint cluster region, *CML* chronic myleloid leukemia, *CRC* colorectal cancer, *EGFR* epidermal growth factor receptor, *EMA* European Medicines Agency, *FDA* Food and Drug Administration, *FLT3* fms like tyrosine kinase 3, *GIST* gastrointestinal stromal tumor, *HNSCC* Head and neck squamous cell carcinoma, *MDS/MPD* myelodysplastic syndrome/ myeloproliferative disorder, *NSCLC* Non-small cell lung carcinoma, *PDAC* pancreatic ductal adenocarcinoma, *PDGFR* platelet derived growth factor receptor, *Ph+* Philadelphia chromosome positive, *p‑mTOR* phosphorylated mammalian target of rapamycin, *RCC* renal cell carcinoma, *RET* rearranged during transfection, *TP53* tumor protein 53, *VEGFR* vascular endothelial growth factor

**Table 3:** Detailed characteristics of the PGC patients (*n* = 10)

Patient number, gender and age	Histological subtype and	Stage and side	Site of metastasis	Tissue tested	Detected mutations by NGS	IHC	Therapy recommendation
1 Female 61 years	Acinic cell carcinoma	IV° Right	Lung	Metastatic	No mutation detected	Not done (due to insufficient tissue material)	No recommendation
2 Male 83 years	Adenocarcinoma	IV° Right	Liver lung	Metastatic (liver)	No mutation detected	EGFR 2+, MET 1+, PDGFRA 1+, PTEN 1+, p‑mTOR 3+, AR 2+	Androgen deprivation therapy
3 Female 37 years	Adenocarcinoma	IV° Right	Bone	Metastatic	No mutation detected	EGFR 2+, KIT 2+, PTEN 2+, p‑mTOR 3+	No recommendation
4 Male 71 years	Adenocarcinoma	IV° Left	Bone	Primary	No mutation detected	EGFR 3+, PTEN 1+, p‑mTOR 1+, AR 2+	Androgen deprivation therapy
5 Male 46 years	Adenoid cystic carcinoma	IV° Left	Lung	Metastatic	No mutation detected	KIT 1+, MET 3+, PDGFR 1+, PTEN 1+, p‑mTOR 2+	Crizotinib
6 Female 56 years	Adenoid cystic carcinoma	IV° Right	Lung	Primary	ATM: exon 32 c.C9142G (p.Leu3048Val)	EGFR 3+, MET 1+, p‑mTOR 1+, PTEN 1+	Cetuximab
7 Male 47 years	Adenoid cystic carcinoma	IV° Right	Lung	Primary	APC: exon 16 c.T3920A (p.I1307K)	KIT 1+, EGFR 2+, MET 1+, PDGFRA 1+, PTEN 1+, p‑mTOR 1+	Imatinib
8 Male 65 years	Carcinoma ex pleomorphic adenoma	IV° Right	Lung, Brain	Metastatic (lung)	TP53 (exon 7): c.C742T (p.R248W)	EGFR 1+, MET 1+, PDGFRA 2+, PTEN 1+, p‑mTOR 1+	Sunitinib
9 Female 67 years	Primary squamous cell carcinoma	IV° Left	Bone, lung	Primary	CDKN2A (exon 2): c.151_155delGTCT (p.V51 fs); FGFR1 (exon 5): c.478_480delGAT (p.Asp.160del); SMARCB1 (exon 9): c.G1130A (p.R3677H)	EGFR 3+, PTEN 2+	Cetuximab
10 Female 72 years	Primary squamous cell carcinoma	IV° Left	Lung	Primary	CDKN2A (exon 2): c.C341T, (p.P114L)	EGFR 1+, MET 3+, PDGFRA 1+, p‑mTOR 1+	Crizotinib

Values in parentheses indicate the immunohistochemical score that was calculated as mentioned in the “Materials and methods” section

*APC* adenomatous polyposis coli, *AR* androgen receptor, *CDKN2A* cyclin-dependent kinase inhibitor 2A, *EGFR* epidermal growth factor receptor, *FiSH* fluorescence in situ hybridization, *PDGFR* platelet derived growth factor receptor, *p‑mTOR* phosphorylated mammalian target of rapamycin, *PTEN* phosphatase and tensin homolog, *TP53* tumor protein 53, *IHC* immunohistochemistry, *NGS* next-generation sequencing

The median turnaround time from the initiation of molecular profiling to therapy initiation was 43 days.

Eventually, three patients received the targeted therapy. One male patient with an adenocarcinoma was administered bicalutamide as ADT but died because of disease progression before restaging was performed. The second patient with a carcinoma ex pleomorphic adenoma received sunitinib 50 mg orally once daily combined with docetaxel every third week but did not respond to this therapy regimen and experienced progressive disease. The third patient had an adenoid cystic carcinoma and was given imatinib 400 mg orally once daily. He achieved a stable disease for 14 months and tolerated the therapy without any treatment-related adverse events.

## Discussion

To our knowledge, this is the first study of individual genomic alterations that have been translated into concrete tailored therapy recommendations in a group of patients with exclusively recurrent, progressive, and therapy-refractory PGC in stage IV in a real-world setting. None of these patients had the histological subtype mucoepidermoid carcinoma (MEC); instead, they had rarer subtypes, making this subgroup analysis even more valuable and unique.

In this retrospective single center subgroup analysis, we exclusively present the molecular profile of all ten patients with PGC. Their disease was relapsed, therapy-refractory and advanced. Tumor tissue was obtained from all patients and characterized regarding molecular profiles. Subsequently, the genomic information of the patients was discussed in a multidisciplinary tumor board (MTB) for PCM to evaluate the possibility of a genomic-based therapy concept that is independent of the tumor’s histological classification (tissue-agnostic drugs).

Tumor samples harbored mutations in *APC, ATM, CDKN2A, FGFR1, SMARCB1, and TP53*. The IHC revealed expressions of EGFR and *p*-mTOR as well as PTEN in eight patients.

Therapeutic options recommended were ADT, cetuximab, crizotinib, sunitinib, and imatinib. Two patients with AR expression were offered ADT to control the disease. Two patients with strong MET expression were suggested crizotinib as a tailored therapy. For two patients with high EGFR expression cetuximab was recommended. Imatinib was considered in one patient due to expression of KIT and PDGFRA. Sunitinib was proposed to one patient because of PDGFRA overexpression. A treatment recommendation was derived for eight patients from the MTB. The drugs were carefully selected for an individualized treatment with special respect to the patient’s clinical and treatment history and concomitant therapies and comorbidities. Interestingly, all these recommendations were based on the protein expressions obtained by immunohistochemistry. Thus, our analysis underscores the clinical relevance of immunohistochemistry in precision medicine.

Eventually, three patients received the targeted therapy. One patient died before restaging was performed. The second patient received sunitinib and did not respond. Imatinib was applied to the third patient who experienced a stable disease for 14 months. Although this analysis showed that PCM is implementable in daily clinical routine, only one patient had a clinical benefit from this therapy approach. One reason may be the turnaround time: a shorter turnaround time may help to start the targeted therapy earlier and to control the cancer disease. Liquid biopsy may be a viable option to reduce the turnaround time, to monitor the disease and to assess the therapy response. Another reason may be the complexity of PGC. The major challenge is the extreme and complex phenotypical, morphological, histological, clinical, and even intertumor and intratumor heterogeneity within the same tumor tissue [45]. The WHO classification of salivary gland tumors 2017 distinguishes over 20 types of malignant salivary gland tumors [4].

The heterogeneity, diversity and the multitude of biological differences between patients may urge the development of novel drugs that are capable of targeting various alterations to increase the efficacy of therapeutic agents and to minimize the risk of drug resistance.

The observed genomic aberrations and overexpression of AR, KIT, and EGFR, PTEN, *p*-mTOR, and PDGFRA in PGC in this analysis are in keeping with previous studies [18–33]. The rationale for the therapy recommendation with ADT was corroborated by a study by Boon et al. They studied the application of ADT in 35 patients with androgen receptor-positive advanced salivary duct carcinoma, which lead to a median overall survival (OS) of 17 months versus 5 months in 43 patients receiving best supportive care [34].

The overexpression of MET was seen in all three patients with adenoid cystic carcinoma and is in line with other studies [35]; however, to our knowledge, this is the first report of an overexpression also in carcinoma ex pleomorphic adenoma and primary squamous cell carcinoma. Crizotinib was offered as a molecularly driven treatment approach. Its clinical efficacy in salivary gland cancers has not yet been described in clinical trials.

Only one study has used molecular profiling to offer an individualized therapy in patients with metastatic salivary gland adenoid cystic carcinoma (ACC). They enrolled a limited 14 patients, of whom 11 actually received the recommended treatment. The investigators reported the clinical benefit of molecularly guided treatment [36]. Imatinib and sunitinib are tyrosine kinase inhibitors that were offered, each in one case, as an alternative therapy in the case of overexpression in PDGFRA/B or KIT. The data pertaining to the use of imatinib in salivary gland cancer are contradictory and unclear. According to two phase II trials that applied imatinib in patients with KIT-positive adenoid cystic cancers of salivary glands, imatinib was not of significant clinical benefit and the best observed response was a stable disease (SD).

As a limitation, however, it should be noted that only ACC was studied, and PDGFR expression of the tumor tissue was not evaluated [37, 38]. In contrast, another phase II trial tested imatinib in 15 patients with ACC of salivary glands and concluded that imatinib was of clinical benefit because it achieved a partial response (PR) in two patients and a SD in five other patients [39]. Likewise, in another study, imatinib achieved significant regression of initially unresectable ACC of salivary glands in two patients, making them eligible for a salvage resection [40].

Similar to imatinib, sunitinib was also tested in a phase II trial in 13 patients with ACC of salivary glands and 11 of these achieved stable disease; however, the investigators did not test the patients’ tumors for KIT or PDGFR expression [41]. Dasatinib is another tyrosine kinase inhibitor that was investigated in a phase II trial for patients with recurrent or metastatic KIT expressing ACC and for nonadenoid cystic malignant salivary tumors. It achieved only one PR in a patient with ACC and the experimental treatment demonstrated no activity in non-ACC salivary gland cancer [42].

In another phase II trial, axitinib was applied in 33 patients with unresectable ACC. Ho et al. reported that axitinib achieved a PR in 3 patients and a SD in 25 patients. The median progression-free survival (PFS) was 5.7 months [43]. Overexpression of EGFR was often observed in salivary gland cancers and provides a solid and sound rationale for the administration of cetuximab [26, 27]. Its clinical efficacy was examined by Locati et al. in 2009 in salivary gland carcinomas, and they reported a clinical benefit rate of 50% [44].

Notably, we identified *p*-mTOR overexpression in eight patients; however, we did not consider *p*-mTOR inhibition with everolimus because of the low evidence for clinical efficacy.

Despite great research efforts and the investigated agents in PGC and other salivary gland cancers, progressive recurrent PGC has a dismal prognosis, and because of the rarity of the disease, well-established therapeutic options are scarce.

Great strides in the in-depth analysis of the vast genetic and epigenetic landscape of salivary gland cancers have been made in recent years; however, the PCM approach remains in its infancy when it comes to implementing novel individualized therapeutic strategies and concepts for this malignancy [33, 45, 46].

It is challenging to classify and prioritize the plethora of reported genetic alterations and epigenetic changes to identify actionable targets and to choose adequate tailored therapeutic measures. Thus, the roles of most identified alterations are undefined regarding pathogenesis, therapeutic consequences, and implications [47].

Another major challenge is the extreme and complex phenotypical, morphological, histological, clinical, and even intertumor and intratumor heterogeneity within the same tumor tissue [45]. The WHO classification of salivary gland tumors 2017 distinguished over 20 types of malignant salivary gland tumors [4]. The heterogeneity, diversity and the multitude of biological differences between patients may urge the development of novel drugs that are capable of targeting various alterations to increase the efficacy of therapeutic agents and to minimize the risk of drug resistance. In light of the complexity of PGC, molecularly driven clinical trials in PCM have to be designed as basket and umbrella trials that take into account the diversity of this malignancy for a better outcome.

## Conclusion

This analysis clearly shows that molecular profiling from tumor samples of patients with advanced, heavily pretreated and therapy-refractory PGC in stage IV is feasible and results in meaningful and rational therapy recommendations and strategies; however, the complex tumor biology, heterogeneity, and extreme rarity remain unique challenges for the management of PGC and need to be addressed by further studies seeking a better understanding of this malignancy. In rare diseases such as PGC where randomized trials cannot be performed easily, molecularly driven treatment approaches and strategies may be particularly useful tools and viable options.

## Abbreviations

ADT: Androgen deprivation therapy
*AKT*: *Alpha serine/threonine-protein kinase*
*ALK*: *Anaplastic lymphoma kinase*
*ALL*: *Acute lymphoblastic leukemia*
*APC*: *Adenomatous polyposis coli*
*AR*: *Androgen receptor*
*ATM*: *Ataxia telangiectasia mutated*
*BAP1*: *BRCA1 associated protein‑1*
*BRAF*: *B‑rapidly accelerated fibrosarcoma*
*BRCA*: *Breast cancer 1*
*CDKN2A*: *Cyclin-dependent kinase inhibitor 2A*
CML: Chronic myeloid leukemia
CRC: Colorectal cancer
DCR: Disease control rate
ECOG: Eastern Cooperative Oncology Group
EGFR: Epidermal growth factor receptor
EMA: European Medicines Agency
FDA: Food and Drug Administration
*FGFR*: *Fibroblast growth factor receptor*
FiSH: Fluorescence in situ hybridization
*FLT3*: *Fms like tyrosine kinase 3*
GIST: Gastrointestinal stromal tumor
HER2: Human epidermal growth factor receptor 2
HNSCC: Head and neck squamous cell carcinoma
HL: Hodgkin lymphoma
*IDH*: *Isocitrate dehydrogenase 1*
IHC: Immunohistochemistry
lossPTEN: Loss of phosphatase and tensin homolog
MDS/MPD: Myelodysplastic syndrome/myeloproliferative disorder
MEC: Mucoepidermoid carcinoma
MTB: Multidisciplinary tumor board
mTOR: Mammalian target of rapamycin
*NF1*: *Neurofibromin 1*
NOS: Not otherwise specified
NSCLC: Non-small cell lung carcinoma
PARP: Poly [ADP-ribose] polymerase 1
PCM: Precision cancer medicine
PDAC: Pancreatic ductal adenocarcinoma
PDGFR: Platelet-derived growth factor receptor
PD-L1: Programmed death-ligand 1
PGC: Parotid gland carcinoma
Ph+: Philadelphia chromosome positive
*PIK3CB*: *Phosphatidylinositol‑4,5‑bisphosphate 3‑kinase catalytic subunit beta*
*p*-mTOR: phosphorylated Mammalian target of rapamycin
PR: Partial response
PTEN: Phosphatase and tensin homolog
*PTPN11*: *Protein phosphatase non-receptor type 11*
*RB1*: *Retinoblastoma 1*
RCC: Renal cell carcinoma
SD: Stable disease
SGC: Salivary gland carcinomas
*SHH*: *Sonic hedgehog*
*SMARCB1*: *SWI/SNF-related matrix-associated actin-dependent regulator of chromatin subfamily B member 1*
*SMO*: *Smoothened*
*STK11*: *Serine/threonine kinase 11*
*TP53*: *Tumor protein 53*
*TRK*: *Neurotrophin receptor kinases*
*VEGFR*: *Vascular endothelial growth factor*
*VHL*: *von Hippel-Lindau*

## References

[1] Holmberg KV, Hoffman MP (2014). Anatomy, biogenesis and regeneration of salivary glands. Monogr Oral Sci.

[2] Amano O, Mizobe K, Bando Y, Sakiyama K (2012). Anatomy and histology of rodent and human major salivary glands: -overview of the Japan salivary gland society-sponsored workshop. Acta Histochem Cytochem.

[3] Chason HM, Downs BW (2018). Anatomy, head and neck, parotid gland.

[4] Seethala RR, Stenman G (2017). Update from the 4th edition of the world health organization classification of head and neck tumours: tumors of the salivary gland. Head Neck Pathol.

[5] Ho K, Lin H, Ann DK, Chu PG, Yen Y (2011). An overview of the rare parotid gland cancer. Head Neck Oncol.

[6] Schwentner I, Obrist P, Thumfart W, Sprinzl G (2006). Distant metastasis of parotid gland tumors. Acta Otolaryngol.

[7] Sood S, McGurk M, Vaz F (2016). Management of salivary gland tumours: United Kingdom national multidisciplinary guidelines. J Laryngol Otol.

[8] Wang X, Luo Y, Li M, Yan H, Sun M, Fan T (2017). Management of salivary gland carcinomas—a review. Oncotarget.

[9] Vander Poorten V, Bradley PJ, Takes RP, Rinaldo A, Woolgar JA, Ferlito A (2012). Diagnosis and management of parotid carcinoma with a special focus on recent advances in molecular biology. Head Neck.

[10] Spiro RH, Huvos AG, Berk R, Strong EW (1978). Mucoepidermoid carcinoma of salivary gland origin. A clinicopathologic study of 367 cases. Am J Surg.

[11] Bhattacharyya N, Fried MP (2005). Determinants of survival in parotid gland carcinoma: a population-based study. Am J Otolaryngol.

[12] Hauschild A, Grob JJ, Demidov LV (2012). Dabrafenib in BRAF-mutated metastatic melanoma: a multicentre, open-label, phase 3 randomised controlled trial. Lancet.

[13] Bang YJ, Van Cutsem E, Feyereislova A (2010). Trastuzumab in combination with chemotherapy versus chemotherapy alone for treatment of HER2-positive advanced gastric or gastro-oesophageal junction cancer (ToGA): a phase 3, open-label, randomised controlled trial. Lancet.

[14] Kantarjian H, Sawyers C, Hochhaus A (2002). Hematologic and cytogenetic responses to imatinib mesylate in chronic myelogenous leukemia. N Engl J Med.

[15] Von Hoff DD, Stephenson JJ, Rosen P (2010). Pilot study using molecular profiling of patients’ tumors to find potential targets and select treatments for their refractory cancers. J Clin Oncol.

[16] Konig IR, Fuchs O, Hansen G, von Mutius E, Kopp MV (2017). Eur Respir J.

[17] Allred DC, Bustamante MA, Daniel CO (1990). Immunocytochemical analysis of estrogen receptors in human breast carcinomas. Evaluation of 130 cases and review of the literature regarding concordance with biochemical assay and clinical relevance. Arch Surg.

[18] Sygut D, Bien S, Ziolkowska M, Sporny S (2008). Immunohistochemical expression of androgen receptor in salivary gland cancers. Pol J Pathol.

[19] Dalin MG, Watson PA, Ho AL, Morris LG (2017). Androgen Receptor Signaling in Salivary Gland Cancer. Cancers (Basel).

[20] Nasser SM, Faquin WC, Dayal Y (2003). Expression of androgen, estrogen, and progesterone receptors in salivary gland tumors. Frequent expression of androgen receptor in a subset of malignant salivary gland tumors. Am J Clin Pathol.

[21] Salehinejad J, Mohtasham N, Bagherpour A, Abbaszadeh-Bidokhty H, Ghazi A (2014). Evaluation of c-kit protein (CD117) expression in common salivary gland neoplasms. J Oral Maxillofac Pathol.

[22] Tariq H, Anjum S, Din HU, Akhtar F (2017). Diagnostic utility of C-kit protein (CD117) expression in differentiating adenoid cystic carcinoma and polymorphous low grade Adenocarcinoma. Pak J Med Sci.

[23] Penner CR, Folpe AL, Budnick SD (2002). C-kit expression distinguishes salivary gland adenoid cystic carcinoma from polymorphous low-grade adenocarcinoma. Mod Pathol.

[24] Sorensen KB, Godballe C, de Stricker K, Krogdahl A (2006). Parotid carcinoma: expression of kit protein and epidermal growth factor receptor. J Oral Pathol Med.

[25] Yamada K, Iwai K, Okada Y, Mori M (1989). Immunohistochemical expression of epidermal growth factor receptor in salivary gland tumours. Virchows Arch A Pathol Anat Histopathol.

[26] Shang J, Shui Y, Sheng L, Wang K, Hu Q, Wei Q (2008). Epidermal growth factor receptor and human epidermal growth receptor 2 expression in parotid mucoepidermoid carcinoma: possible implications for targeted therapy. Oncol Rep.

[27] Clauditz TS, Gontarewicz A, Lebok P (2012). Epidermal growth factor receptor (EGFR) in salivary gland carcinomas: potentials as therapeutic target. Oral Oncol.

[28] Liu H, Du L, Wang R (2015). High frequency of loss of PTEN expression in human solid salivary adenoid cystic carcinoma and its implication for targeted therapy. Oncotarget.

[29] Ettl T, Baader K, Stiegler C (2012). Loss of PTEN is associated with elevated EGFR and HER2 expression and worse prognosis in salivary gland cancer. Br J Cancer.

[30] Clauditz TS, Gontarewicz A, Bokemeyer C (2013). Abundant expression of mTOR kinase in salivary gland tumors—potentials as therapy target?. J Oral Pathol Med.

[31] Ettl T, Schwarz-Furlan S, Haubner F (2012). The PI3K/AKT/mTOR signalling pathway is active in salivary gland cancer and implies different functions and prognoses depending on cell localisation. Oral Oncol.

[32] Yamamoto S, Fukumoto E, Yoshizaki K (2008). Platelet-derived growth factor receptor regulates salivary gland morphogenesis via fibroblast growth factor expression. J Biol Chem.

[33] Kato S, Elkin SK, Schwaederle M (2015). Genomic landscape of salivary gland tumors. Oncotarget.

[34] Boon E, van Boxtel W, Buter J (2018). Androgen deprivation therapy for androgen receptor-positive advanced salivary duct carcinoma: a nationwide case series of 35 patients in the Netherlands. Head Neck.

[35] Bell D, Ferrarotto R, Fox MD (2015). Analysis and significance of c-MET expression in adenoid cystic carcinoma of the salivary gland. Cancer Biol Ther.

[36] Popovtzer A, Sarfaty M, Limon D (2015). Metastatic salivary gland tumors: a single-center study demonstrating the feasibility and potential clinical benefit of molecular-profiling-guided therapy. Biomed Res Int.

[37] Pfeffer MR, Talmi Y, Catane R, Symon Z, Yosepovitch A, Levitt M (2007). A phase II study of Imatinib for advanced adenoid cystic carcinoma of head and neck salivary glands. Oral Oncol.

[38] Hotte SJ, Winquist EW, Lamont E (2005). Imatinib mesylate in patients with adenoid cystic cancers of the salivary glands expressing c-kit: a Princess Margaret Hospital phase II consortium study. J Clin Oncol.

[39] Guigay JM, Bidault F, Temam S (2007). Antitumor activity of imatinib in progressive, highly expressing KIT adenoid cystic carcinoma of the salivary glands: A phase II study. J Clin Oncol..

[40] Alcedo JC, Fabrega JM, Arosemena JR, Urrutia A (2004). Imatinib mesylate as treatment for adenoid cystic carcinoma of the salivary glands: report of two successfully treated cases. Head Neck.

[41] Chau NG, Hotte SJ, Chen EX (2012). A phase II study of sunitinib in recurrent and/or metastatic adenoid cystic carcinoma (ACC) of the salivary glands: current progress and challenges in evaluating molecularly targeted agents in ACC. Ann Oncol.

[42] Wong SJ, Karrison T, Hayes DN (2016). Phase II trial of dasatinib for recurrent or metastatic c-KIT expressing adenoid cystic carcinoma and for nonadenoid cystic malignant salivary tumors. Ann Oncol.

[43] Ho AL, Dunn L, Sherman EJ (2016). A phase II study of axitinib (AG-013736) in patients with incurable adenoid cystic carcinoma. Ann Oncol.

[44] Locati LD, Bossi P, Perrone F (2009). Cetuximab in recurrent and/or metastatic salivary gland carcinomas: A phase II study. Oral Oncol.

[45] Grunewald I, Vollbrecht C, Meinrath J, Wardelmann E (2015). Targeted next generation sequencing of parotid gland cancer uncovers genetic heterogeneity. Oncotarget.

[46] Seethala RR, Griffith CC (2016). Molecular pathology: predictive, prognostic, and diagnostic markers in salivary gland tumors. Surg Pathol Clin.

[47] Seymour CW, Gomez H, Chang CH (2017). Precision medicine for all? Challenges and opportunities for a precision medicine approach to critical illness. Crit Care.

